# Centromere protein U (CENPU) promotes gastric cancer cell proliferation and glycolysis by regulating high mobility group box 2 (HMGB2)

**DOI:** 10.1080/21655979.2021.2002018

**Published:** 2021-12-07

**Authors:** Taozhi Deng, Xuemei Jiang, Zhoutao He, Manni Cai, Chaochao Chen, Zewen Xu

**Affiliations:** a Department of Gastroenterology, Hainan Cancer Hospital, Haikou, Hainan Province, China; b Department of Gastroenterology, Hainan General Hospital, Haikou, Hainan Province, China

**Keywords:** CENPU, HMGB2, glycolysis, gastric cancer

## Abstract

Gastric cancer is one of the most common malignancy with a leading mortality rate worldwide. Despite the progress in the diagnosis and therapeutic strategy, the associated mortality is still growing. It is of great significance to understand molecular mechanisms of the development of gastric cancer. Glycolysis is a main source of ATP provision for cancer cells including gastric cancer, and targeting glycolysis is a promising therapeutic strategy. Centromere protein U (CENPU) has been found to be overexpressed in many types of cancer. Downregulation of CENPU suppresses the proliferation and invasion of cancer cells. High mobility group box 2 (HMGB2) is identified as a biomarker to diagnose of gastric cancer. Knockdown of HMGB2 inhibits proliferation and glycolysis in gastric cancer cells. In this work, we identified that CENPU was upregulated in gastric cancer. Knockdown of CENPU was able to suppress the proliferation and glycolysis of gastric cancer cells. Further the results showed that the anti-cancer effect of CENPU was HMGB2-dependent. Taken together, CENPU is an upstream factor of HMGB2, which regulates proliferation and glycolysis of gastric cancer.

## Introduction

Gastric cancer is a most common malignancy and ranks as the second lethality worldwide [1–3]. Despite advances in the diagnosis and therapeutic strategy, the mortality rate caused by gastric cancer is still growing [4]. Thus, it is urgent and significant to develop new therapeutic targets and strategies to fight against cancer. Metabolic plasticity allows tumor cells to reprogram metabolism pathway to cope with tumor microenvironment, which is a hallmark of cancer [5]. Cancer cells undergo enhanced glycolysis even in presence of oxygen, and this phenomenon was named ‘Warburg effect’ [6]. Glycolytic metabolism provides substrates for macromolecular synthesis and leads to acidification of the microenvironment, thereby allowing for enhanced invasiveness [7,8]. Under hypoxic conditions, glycolysis is the predominant pathway producing ATP, and targeting glycolysis remains attractive for cancer therapy [9].

High mobility group box (HMGB) is ubiquitous and abundant nonhistone nuclear protein [10,11]. HMGB family bends DNA, and subsequently modulates DNA transcription, replication, repair, and recombination [11]. HMGB family consists of HMGB1, HMGB2, HMGB3 and HMGB4 [11]. HMGB2 facilitates carcinogenesis and is a novel biomarker for the diagnosis of gastric cancer [12]. Knockdown of HMGB2 significantly suppressed proliferation, invasion, and glycolysis of gastric cancer cells [12].

Centromere protein U (CENPU), is a member of CENP family [13,14]. CENPU is a constituent of centromere, which is necessary for the recovery from spindle damage [13]. CENPU is also a cellular transcriptional repressor [15]. CENPU has been found to be overexpressed in a variety of tumor tissues and cancer phenotypes, and high expression level of CENPU predicts poor prognosis in many cancer phenotypes [13]. Suppression of CENPU expression inhibits cancer cell proliferation, migration, and invasion [13,14].

HMGB2 is identified as a downstream factor of CENPU, and CENPU enhances the expression level of HMGB2 in ovarian cancer cells [16]. CENPU facilitates aggressiveness ability and progression of ovarian cancer via HMGB2 [16]. In this work, we aimed to investigate the regulatory role of CENPU in gastric cancer and its correlation with HMGB2, and their effects on glycolytic metabolism. TCGA shows that CENPU is upregulated in gastric cancer, but its biological function and mechanism are still not clear. This study demonstrated that downregulation of CENPU inhibited cell proliferation and glycolysis of gastric cancer by regulating HMGB2.

## Materials and methods

### Human cell lines and reagents

GES-1, AGS, MKN45 and HGC-27 cell lines were purchased from American Type Culture Collection (ATCC, USA). Fetal bovine serum (FBS), DMEM medium and trypsin-EDTA were provided by Thermo Fisher Scientific (CN, China). Cells were maintained in DMEM supplemented with 10% FBS, 100 IU/ml penicillin, 100 IU/ml streptomycin in 37°C, 5% CO_2_ humidified incubator. Primary antibodies against CENPU (AB_2639655), HMGB2 (AB_2642476) were purchased from Thermo Fisher Scientific (CN, China). Primary antibody against LDHA (#2012S) was purchased from Cell Signaling Technology (CST). Primary antibodies against GLUT1 (ab14683), HK2 (ab227198), ki-67 (ab833), GAPDH (ab9485) and secondary antibody horseradish peroxidase (HRP) were supplied by Abcam. Glucose Assay Kit, Lactate Assay Kit II, and ATP Colorimetric Assay Kit were purchased from BioVision (Milpitas, CA).

### Quantitative reverse transcription polymerase chain reaction (qRT-PCR)

Total RNA extraction and qRT-PCR for quantification of CENPU mRNA level were performed as previously described [17,18]. The CENPU transcript level was calculated with 2^−ΔΔCt^ method [19]. GAPDH served as an internal control. The primers used for PCR reaction were as following: GAPDH-forward: 5ʹ-GGAGCGAGATCCCTCCAAAAT-3ʹ and GAPDH-reverse: 3ʹ-GGCTGTTGTCATACTTCTCATGG-5ʹ; CENPU-forward: 5ʹ-ACCCACCTAGAGCATCAACAA-3ʹ and CENPU-reverse: 3ʹ-ACTTCAATCATACGCTGCCTTT-5ʹ.

### Cell viability assay

MTT assay was used to detect the cell viability. Cells were seeded in a 96-well plate at a cell density of 1 × 10^5^/ml. The next day, plasmid transfection was performed with a polyethylenimine (PEI)-mediated transfection method. OD value was measured at 570 nm with a plate reader.

### Colony formation assay

Rich DMEM culture medium supplemented with 0.75% agar, or 0.36% agar was used for the base agar layer or for the top layer, respectively. Cells after transfection with plasmid were resuspended at a final concentration of 3 × 10^4^ cells/ml and cultured for 3 weeks. Colonies were stained with 0.04% crystal violet in PBS containing 2% ethanol. After washed with PBS and dried in the air, images of stained colonies were taken with a scanner.

### Glycolysis measurement

Glucose consumption, lactate production and total ATP content were assessed with Glucose Assay Kit, Lactate Assay Kit and ATP Assay Kit, respectively, according to the manufacturer’s instructions.

### Western blotting

Cellular protein extracts were boiled and separated by SDS-PAGE gel electrophoreses. The bands were blotted via immunoblotting and visualized with enhanced chemiluminescence using the Odyssey system. The images were analyzed with ImageJ software. GAPDH served as the internal control.

### Immunohistochemistry assay

Tumor tissues were embedded with paraffin, deparaffinized with xylene, hydrated with ethanol. And then the tissues were washed with PBS and neutralized with 1% hydrogen peroxidase at 37°C for 30 min. Then tissues were incubated with the indicated primary antibodies for 90 min at room temperature. The tissues were washed with PBS and incubated with secondary antibody for 45 min. Tissues were then stained with 3,3-diaminobenzidine and counterstained with hematoxylin. At last, tissues were dehydrated and detected with an inverted optical microscope.

### Xenograft tumor model

Cells were detached by trypsin-EDTA and washed twice with PBS. Cells were resuspended in 300 µl PBS contains 3 × 10^6^ cells and injected into 5 weeks old mouse. Each group had 3 mice. The growth of tumor was observed and the tumor diameters were measured with digital callipers. All animal experiments were approved by the Ethics Committee of Hainan General Hospital for the use of animals and conducted in accordance with the National Institutes of Health Laboratory Animal Care and Use Guidelines.

### Statistical analysis

Data were analyzed with GraphPad and presented as mean ± SEM. Student’s *t*-test or one-way analysis of variance (ANOVA) was used to qcompare between groups. *p* < 0.05 was considered as statistically significant.

## Results

### CENPU was upregulated in gastric cancer

**Figure 1.**
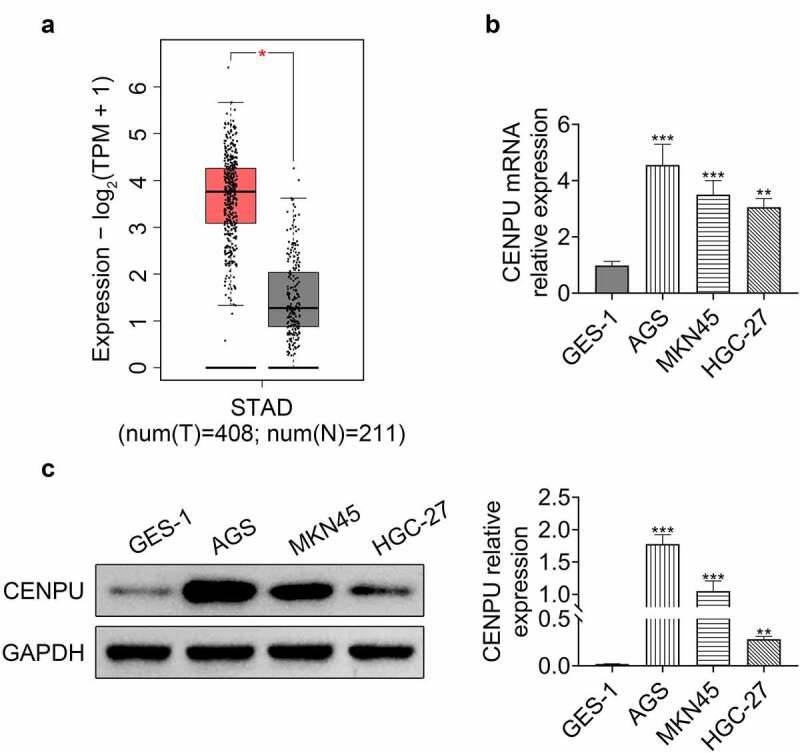
CENPU was upregulated in gastric cancer. (a) GEPIA showed that CENPU was upregulated in gastric cancer cells. (b) qPCR was used to detect the relative expression level of CENPU in human gastric epithelial cell line GES-1, and in gastric cancer cell lines AGS, MKN45, HGC-27. **p < 0.01 *vs* GES-1, ***p < 0.001 *vs* GES-1. Data are mean ± SEM of at least three independent experiments. (c) *Left*, representative blots of WB analysis of CENPU protein expression level in human gastric epithelial cell line GES-1, and in gastric cancer cell lines AGS, MKN45, HGC-27. *Right*, the relative protein expression level of CENPU normalized to GAPDH. **p < 0.01 *vs* GES-1, ***p < 0.001 *vs* GES-1. Data are mean ± SEM of at least three independent experiments

First, we checked the expression level of CENPU in gastric cancer. GEPIA (Gene Expression Profiling Interactive Analysis) revealed that CENPU was upregulated in gastric cancer cells (Figure 1a). qPCR and Western blotting analysis showed that in gastric cancer cell lines AGS, MKN45 and HGC-27, the mRNA level and the relative protein expression level of CENPU were significantly increased, compared with human gastric epithelial cell line GES-1 (Figures 1a and c). These results demonstrated that CENPU was upregulated in gastric cancer.

### Knockdown of CENPU inhibited proliferation of gastric cancer cells

**Figure 2.**
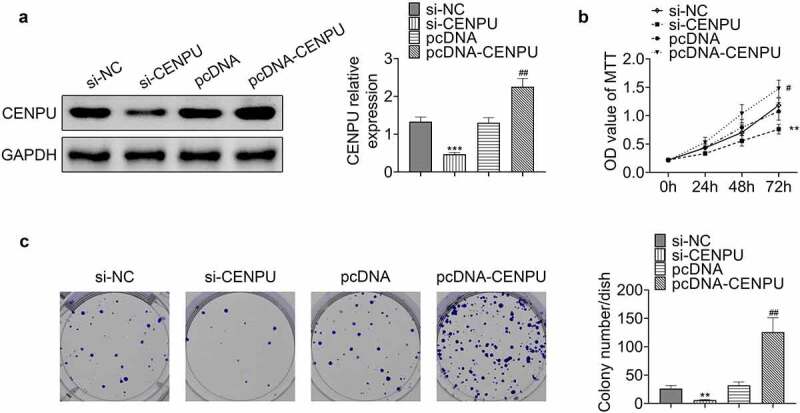
Knockdown of CENPU inhibited proliferation of gastric cancer cells. (a) *Left*, representative blots of WB analysis of CENPU protein expression level after transfections with si-CENPU and pcDNA-CENPU. *Right*, the relative protein expression level of CENPU normalized to GAPDH. ***p < 0.001 *vs* si-NC, ^##^*p* < 0.01 *vs* pcDNA. Data are mean ± SEM of at least three independent experiments. (b) MTT assay was used to assess the cell proliferation after transfection with si-CENPU and pcDNA-CENPU in AGS cells. **p < 0.01 *vs* si-NC, ^#^*p* < 0.05 *vs* pcDNA. Data are mean ± SEM of at least three independent experiments. (c) *Left*, representative images of cell colony formation after transfections with si-CENPU and pcDNA-CENPU. *Right*, the average number of colonies in each dish of at least three independent experiments after transfection with si-CENPU and pcDNA-CENPU in AGS cells. **p < 0.01 *vs* si-NC, ^##^*p* < 0.01 *vs* pcDNA

To investigate the regulatory role of CENPU in proliferation of gastric cancer cells, we used siRNA gene silencing and detected cell proliferation in AGS cell line. siRNA vector targeting CENPU and plasmid carrying CENPU gene were generated to knockdown and overexpress CENPU, respectively (Figure 2a). Knockdown of CENPU inhibited proliferation of AGS cells, and overexpression of CENPU enhanced growth of AGS cells (Figure 2b). Knockdown of CENPU suppressed colony formation, and overexpression of CENPU dramatically promoted colony formation of AGS cells (Figure 2c). These observations indicated that knockdown of CENPU inhibited cell proliferation and CENPU overexpression promoted cell growth in gastric cancer cells.

### Knockdown of CENPU inhibited glycolysis of gastric cancer cells

**Figure 3.**
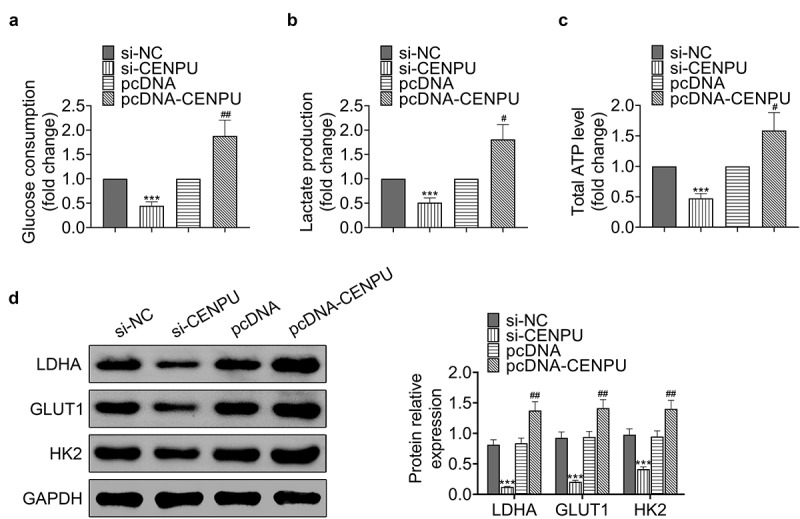
Knockdown of CENPU inhibited glycolysis of gastric cancer cells. (a) Glucose consumption was detected by glucose assay kit in AGS cells. ***p < 0.001 *vs* si-NC, ^##^*p* < 0.01 *vs* pcDNA. Data are mean ± SEM of at least three independent experiments. (b) Lactate production was assessed by lactate assay kit in AGS cells. ***p < 0.001 *vs* si-NC, ^#^*p* < 0.05 *vs* pcDNA. Data are mean ± SEM of at least three independent experiments. (c) Total ATP content was measured by ATP assay kit. ***p < 0.001 *vs* si-NC, ^#^*p* < 0.05 *vs* pcDNA. Data are mean ± SEM of at least three independent experiments. (d) *Left*, representative blots of WB analysis of LDHA, GLUT1 and HK2 protein expression levels after transfections with si-CENPU and pcDNA-CENPU. *Right*, the relative protein expression levels of LDHA, GLUT1 and HK2 normalized to GAPDH. ***p < 0.001 *vs* si-NC, ^##^*p* < 0.01 *vs* pcDNA. Data are mean ± SEM of at least three independent experiments

Then we explore whether CENPU affects glycolytic metabolism of gastric cells. Knockdown of CENPU significantly decreased glucose consumption, lactate production and ATP generation, which were obviously enhanced by CENPU overexpression in AGS cells (Figure 3a-c). Knockdown of CENPU significantly decreased the protein expression levels of LDHA, GLUT1 and HK2, which were enhanced by CENPU overexpression in AGS cells (Figure 3d). These results demonstrated that CENPU knockdown suppressed the glycolysis and CENPU overexpression promoted the glycolysis of gastric cancer cells.

### CENPU promoted proliferation and glycolysis of gastric cancer cells via HMGB2

**Figure 4.**
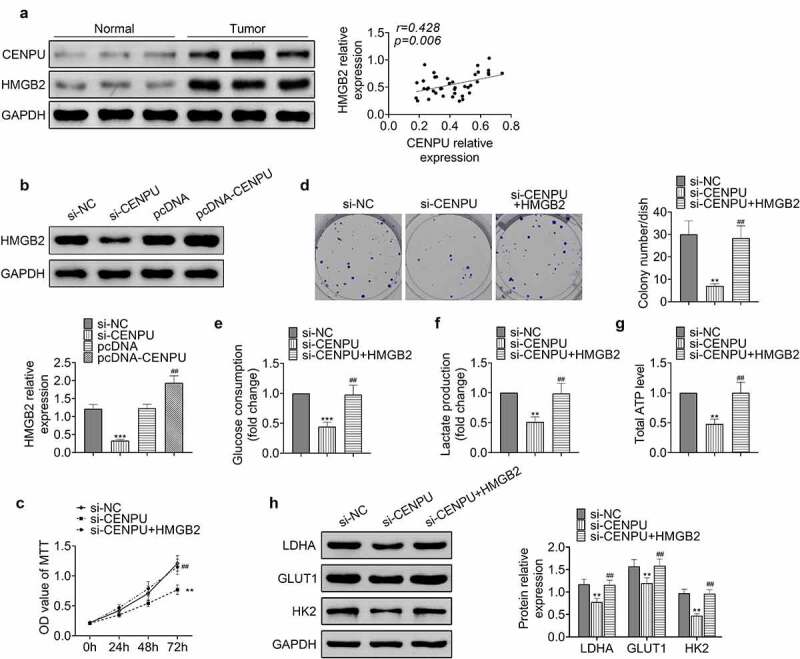
CENPU promoted proliferation and glycolysis of gastric cancer cells via HMGB2. (a) *Left*, representative blots of lysate of normal tissue adjacent to the tumor and gastric cancer tissue from gastric cancer patient. n = 40. *Right*, correlation analysis of the protein expression levels of CENPU and HMGB2. (b) *Upper*, representative blots of WB analysis of HMGB2 protein expression level after transfections with si-CENPU and pcDNA-CENPU. *Lower*, the relative protein expression level of HMGB2 normalized to GAPDH. ***p < 0.001 *vs* si-NC, ^##^*p* < 0.01 *vs* pcDNA. Data are mean ± SEM of at least three independent experiments. (c) MTT assay was used to assess the cell proliferation after transfection with si-CENPU and pcDNA-HMGB2 in AGS cells. **p < 0.01 *vs* si-NC, ^##^*p* < 0.01 *vs* si-CENPU. Data are mean ± SEM of at least three independent experiments. (d) *Left*, representative images of cell colony formation after transfections with si-CENPU and pcDNA-HMGB2 in AGS cells. *Right*, the average number of colonies in each dish of at least three independent experiments after transfection with si-CENPU and pcDNA-CENPU in AGS cells. **p < 0.01 *vs* si-NC, ^##^*p* < 0.01 *vs* si-CENPU. (e) Glucose consumption was detected by glucose assay kit in AGS cells. ***p < 0.001 *vs* si-NC, ^##^*p* < 0.01 *vs* si-CENPU. Data are mean ± SEM of at least three independent experiments. (f) Lactate production was assessed by lactate assay kit in AGS cells. **p < 0.01 *vs* si-NC, ^##^*p* < 0.01 *vs* si-CENPU. Data are mean ± SEM of at least three independent experiments. (g) Total ATP content was measured by ATP assay kit. **p < 0.01 *vs* si-NC, ^##^*p* < 0.01 *vs* si-CENPU. Data are mean ± SEM of at least three independent experiments. (h) *Left*, representative blots of WB analysis of LDHA, GLUT1 and HK2 protein expression levels after transfections with si-CENPU and pcDNA-HMGB2 in AGS cells. *Right*, the relative protein expression levels of LDHA, GLUT1 and HK2 normalized to GAPDH. **p < 0.01 *vs* si-NC, ^##^*p* < 0.01 *vs* si-CENPU. Data are mean ± SEM of at least three independent experiments

Next the effect of CENPU on the expression level of HMGB2 and their roles in proliferation and glycolysis in gastric cancer cells were investigated. The protein expression levels of CENPU and HMGB2 exhibited positive correlation (Figure 4a). Knockdown of CENPU caused decreased expression level of HMGB2, and overexpression of CENPU led to enhanced expression level of HMGB2 in AGS cells (Figure 4b). Knockdown of CENPU inhibited proliferation and colony formation of AGS cells, which could be rescued by overexpression of HMGB2 (Figures 4c and d). Knockdown of CENPU significantly suppressed glucose consumption, lactate production and ATP generation in AGS cells, which were rescued by overexpression of HMGB2 (Figure 4e-g). Knockdown of CENPU caused decreased expression levels of LDHA, GLUT1 and HK2 in AGS cells, which were rescued by overexpression of HMGB2 (Figure 4h). These data indicated that CENPU enhanced proliferation and glycolysis of gastric cancer cells via HMGB2.

### CENPU promoted tumor growth in vivo

**Figure 5.**
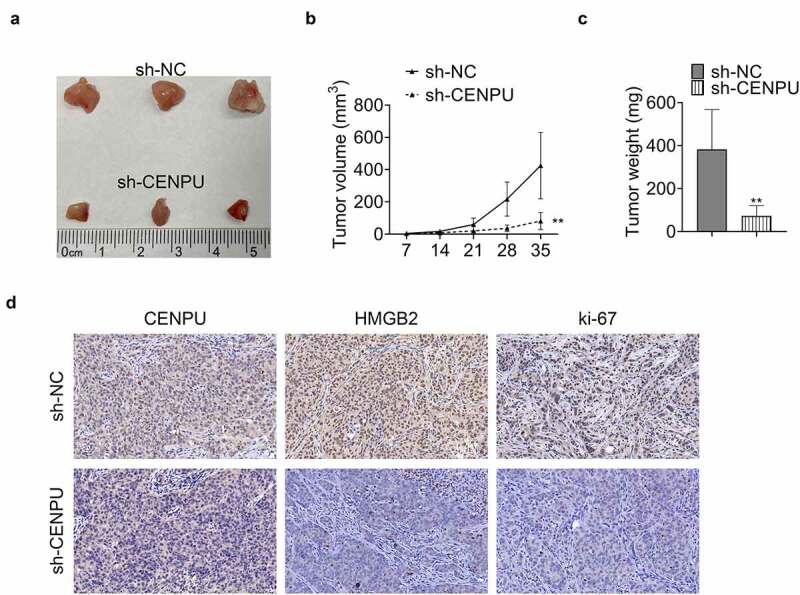
CENPU promoted tumor growth *in vivo*. (a) Representative image of tumor tissue size from xenograft mouse model. (b) Tumor volume measured on indicated days. **p < 0.01 *vs* sh-NC, Data are mean ± SEM of tumors from three mice. (c) Tumor weight measured on thirty-fifth day. **p < 0.01 *vs* sh-NC, Data are mean ± SEM of tumors from five mice. (d) Representative image of IHC assay, which was used to detect the expression levels of CENPU, HMGB2 and ki-67 in tumor tissue

In the last, the influence of CENPU on the tumor growth *in vivo* was investigated. Knockdown of CENPU suppressed the tumor growth with significantly decreased tumor volume and tumor weight (Figure 5a-c). IHC assay revealed that knockdown of CENPU led to significantly deceased expression levels of CENPU, HMGB2 and ki-67 in tumor tissue (Figure 5d). These results showed that CENPU promoted the growth of tumor *in vivo*.

## Discussion

Gastric cancer is one of the most lethal malignancies worldwide [1–3]. However, gastric cancer is likely to become chemo-resistant and remains high lethality [4,20]. New therapeutic targets and strategies are required to combat cancer including gastric cancer. Therefore, it is of great significance to identify the molecular mechanisms of the development of gastric cancer. In this work, we identified that CENPU is upstream of HMGB2, which regulated proliferation and glycolysis of gastric cancer. CENPU may act as a biomarker for the diagnosis and therapeutic target for the treatment of gastric cancer.

CENPU is overexpressed in many cancer phenotypes, and regulates tumor development [13]. Knockdown of CENPU inhibits cancer cell proliferation and invasion [13,14]. HMGB promoted carcinogenesis and is a novel biomarker for the diagnosis of gastric cancer [12]. Knockdown of HMGB2 inhibits proliferation and glycolysis of gastric cancer cells [12]. HMGB2 is found to be downstream of CENPU, and CENPU promoted malignancy and development of ovarian cancer via HMGB2 [16]. Ki-67 protein, whose expression is strictly associated with cell proliferation, has been extensively used as a proliferation marker [21].

Metabolic switch is a hallmark of cancer, allowing cancer cells to adapt to microenvironment change [5,6]. Glycolytic metabolism is a main source of ATP provision for cancer cells, and glycolysis provides metabolites and acidic microenvironment that favors malignant development of tumor [7,8]. Thus, cancer cells display many metabolic phenotypes, such as glucose consumption, lactate generation, ATP production, extracellular acidification to support biosynthesis, proliferation, differentiation, migration and invasion [22]. Accumulating evidences demonstrate that glycolysis plays a key role in tumorigenesis of different cancer types including gastric cancer [23]. It has been realized that glucose metabolism and glycolytic activity are the main drivers of carcinogenesis [23]. Glucose transports (GLUTs) are overexpressed in most cancer cells to satisfy their increased glycolysis rates [24]. GLUT1, encoded by SLC2A1, is important in cancer development and is associated with poor prognosis in many cancers [24,25]. Inhibition of GLUT1 expression reverses Warburg effect and induces apoptosis of gastric cancer cells [24]. Hexokinases (HKs) irreversibly convert glucose to glucose-6-phosphate (G-6P) at the first step of glucose metabolism [26]. Among all HKs, HK2 is a major rate-limiting enzyme of glycolysis, and upregulation of HK2 contributes to enhanced glycolysis [26,27]. HK2 is abnormally expressed in a variety of cancers including gastric cancer, and the expression level of HK2 is closely associated with cancer prognosis and mortality [26]. LDHA is a key glycolytic enzyme that catalyzes the reversible interconversion between L-lactate and pyruvate at the last step of glycolysis [28]. Inhibition of LDHA expression diminishes tumorigenesis, increases mitochondrial oxidative respiration, and decreases glycolysis [28].

In the present study, we demonstrated that CENPU promoted proliferation and glycolysis of gastric cancer cells via HMGB2. CENPU was upregulated in gastric cancer. Inhibition of CENPU expression suppressed proliferation and glycolysis of gastric cancer cells. CENPU was an upstream protein of HMGB2, and CENPU enhances the expression level of HMGB2 in gastric cancer cells. Thus, targeting CENPU is a promising therapeutic strategy for treatment of gastric cancer.

## Conclusion

We showed that CENPU was upregulated in gastric cancer cell lines. Knockdown of CENPU by siRNA gene silencing suppressed the proliferation and glycolysis activity of AGS gastric cancer cells. We further demonstrated that the anti-cancer effect of CENPU exhibited a HMGB2-dependent manner. In summary, CENPU was an upstream protein of HMGB2, which regulated proliferation and glycolysis of gastric cancer.

## Highlight

- CENPU was upregulated in gastric cancer
  Knockdown of CENPU inhibited proliferation of gastric cancer cells
- Knockdown of CENPU inhibited glycolysis of gastric cancer cells
- CENPU promoted proliferation and glycolysis of gastric cancer cells via HMGB2
- CENPU promoted tumor growth in vivo

## References

[1] Jemal A, Center MM, DeSantis C, et al. Global patterns of cancer incidence and mortality rates and trends. Cancer Epidemiol Biomarkers Prev. 2010;19(8):1893–1907. 20647400 10.1158/1055-9965.EPI-10-0437

[2] Karimi P, Islami F, Anandasabapathy S, et al. Gastric cancer: descriptive epidemiology, risk factors, screening, and prevention. Cancer Epidemiol Biomarkers Prev. 2014;23(5):700–713. 24618998 10.1158/1055-9965.EPI-13-1057 PMC4019373

[3] Den Hoed CM, Kuipers EJ. Gastric cancer: how can we reduce the incidence of this disease? Curr Gastroenterol Rep. 2016;18(7):34. 27184043 10.1007/s11894-016-0506-0 PMC4868864

[4] Zhang B, Wu J, Cai Y, et al. AAED1 modulates proliferation and glycolysis in gastric cancer. Oncol Rep. 2018;40(2):1156–1164. 29901208 10.3892/or.2018.6478

[5] Guo L. Mitochondria and the permeability transition pore in cancer metabolic reprogramming. Biochem Pharmacol. 2021;188:114537. 33811907 10.1016/j.bcp.2021.114537

[6] Yang H, Li Y, Hu B. Potential role of mitochondria in gastric cancer detection: fission and glycolysis. Oncol Lett. 2021;21(6):439. 33868477 10.3892/ol.2021.12700 PMC8045152

[7] Liberti MV, Locasale JW. The warburg effect: how does it benefit cancer cells? Trends Biochem Sci. 2016;41(3):211–218. 26778478 10.1016/j.tibs.2015.12.001 PMC4783224

[8] Hsu PP, Sabatini DM. Cancer cell metabolism: warburg and beyond. Cell. 2008;134(5):703–707. 18775299 10.1016/j.cell.2008.08.021

[9] Ganapathy-Kanniappan S, Geschwind J-FH. Tumor glycolysis as a target for cancer therapy: progress and prospects. Mol Cancer. 2013;12(1):1–11. 24298908 10.1186/1476-4598-12-152 PMC4223729

[10] Pallier C, Scaffidi P, Chopineau-Proust S, et al. Association of chromatin proteins high mobility group box (HMGB) 1 and HMGB2 with mitotic chromosomes. Mol Biol Cell. 2003;14(8):3414–3426. 12925773 10.1091/mbc.E02-09-0581 PMC181577

[11] Han X, Zhong S, Zhang P, et al. Identification of differentially expressed proteins and clinicopathological significance of HMGB2 in cervical cancer. Clin Proteomics. 2021;18(1):2. 33407071 10.1186/s12014-020-09308-4 PMC7789524

[12] Cui G, Cai F, Ding Z, et al. HMGB2 promotes the malignancy of human gastric cancer and indicates poor survival outcome. Hum Pathol. 2019;84:133–141. 30296520 10.1016/j.humpath.2018.09.017

[13] Wang X, Chen D, Gao J, et al. Centromere protein U expression promotes non-small-cell lung cancer cell proliferation through FOXM1 and predicts poor survival. Cancer Manag Res. 2018;10:6971–6984. 30588102 10.2147/CMAR.S182852 PMC6298391

[14] Li J, Wang ZG, Pang LB, et al. Reduced CENPU expression inhibits lung adenocarcinoma cell proliferation and migration through PI3K/AKT signaling. Biosci Biotechnol Biochem. 2019;83(6):1077–1084. 30849291 10.1080/09168451.2019.1588094

[15] Pan H-Y, Zhang Y-J, Wang X-P, et al. Identification of a novel cellular transcriptional repressor interacting with the latent nuclear antigen of Kaposi’s sarcoma-associated herpesvirus. J Virol. 2003;77(18):9758–9768. 12941884 10.1128/JVI.77.18.9758-9768.2003 PMC224565

[16] Li H, Zhang H, Wang Y. Centromere protein U facilitates metastasis of ovarian cancer cells by targeting high mobility group box 2 expression. Am J Cancer Res. 2018;8(5):835. 29888106 PMC5992511

[17] Zhang H, Tang J, Li C, et al. MiR-22 regulates 5-FU sensitivity by inhibiting autophagy and promoting apoptosis in colorectal cancer cells. Cancer Lett. 2015;356(2Pt B):781–790. 25449431 10.1016/j.canlet.2014.10.029

[18] Caracciolo D, Di Martino MT, Amodio N, et al. miR-22 suppresses DNA ligase III addiction in multiple myeloma. Leukemia. 2019;33(2):487–498. 30120376 10.1038/s41375-018-0238-2 PMC6365379

[19] Schmittgen TD, Livak KJ. Analyzing real-time PCR data by the comparative CT method. Nat Protoc. 2008;3(6):1101–1108. 18546601 10.1038/nprot.2008.73

[20] Re-assembled Casein Micelles for Oral Delivery of Chemotherapeutic Combinations to Overcome Multidrug Resistance in Gastric Cancer. J Mol Clin Med. 2018;1 2

[21] Scholzen T, Gerdes J. The Ki‐67 protein: from the known and the unknown. J Cell Physiol. 2000;182(3):311–322. 10653597 10.1002/(SICI)1097-4652(200003)182:3<311::AID-JCP1>3.0.CO;2-9

[22] Agathocleous M, Harris WA. Metabolism in physiological cell proliferation and differentiation. Trends Cell Biol. 2013;23(10):484–492. 23756093 10.1016/j.tcb.2013.05.004

[23] Liu Y, Zhang Z, Wang J, et al. Metabolic reprogramming results in abnormal glycolysis in gastric cancer: a review. Onco Targets Ther. 2019;12:1195–1204. 30863087 10.2147/OTT.S189687 PMC6389007

[24] Zhang T-B, Zhao Y, Tong Z-X, et al. Inhibition of glucose-transporter 1 (GLUT-1) expression reversed Warburg effect in gastric cancer cell MKN45. Int J Clin Exp Med. 2015;8(2):2423. 25932183 PMC4402830

[25] Ding N, Xu S, Zheng S, et al. “Sweet tooth”-oriented SN38 prodrug delivery nanoplatform for targeted gastric cancer therapy. J Mat Chem B. 2021;9(12):2816–2830. 10.1039/d0tb02787a 33690741

[26] Wu J, Zhang X, Wang Y, et al. Licochalcone A suppresses hexokinase 2-mediated tumor glycolysis in gastric cancer via downregulation of the Akt signaling pathway. Oncol Rep. 2018;39(3):1181–1190. 29286170 10.3892/or.2017.6155

[27] Shao M, Zhang J, Zhang J, et al. SALL4 promotes gastric cancer progression via hexokinase II mediated glycolysis. Cancer Cell Int. 2020;20(1):188. 32489324 10.1186/s12935-020-01275-y PMC7247129

[28] Cui Y, Qin L, Wu J, et al. SIRT3 enhances glycolysis and proliferation in sirt3-expressing gastric cancer cells. PLoS One. 2015;10(6):e0129834. 26121691 10.1371/journal.pone.0129834 PMC4487898

